# Persistent Sleepiness After Effective Positive Airway Pressure Therapy Deserves Independent Clinical Evaluation

**DOI:** 10.7759/cureus.118303

**Published:** 2026-10-21

**Authors:** Binh Pham

**Affiliations:** 1 Psychiatry, Western University of Health Sciences, Pomona, USA; 2 Sleep Medicine, Amen Clinics, Costa Mesa, USA; 3 Sleep Medicine, Southwest Healthcare Medical Education Consortium, Temecula, USA

**Keywords:** central disorders of hypersomnolence, circadian misalignment, editorial, excessive daytime sleepiness, insufficient sleep, obstructive sleep apnea, positive airway pressure therapy, sedating medications, treatment adherence, treatment efficacy

## Abstract

Positive airway pressure (PAP) therapy often improves excessive daytime sleepiness in obstructive sleep apnea (OSA), yet effective control of sleep-disordered breathing does not invariably restore normal daytime alertness. Persistent sleepiness after PAP therapy should therefore be regarded as a clinical observation requiring explanation rather than automatically attributed to treated OSA. The term “residual excessive daytime sleepiness” may become misleading when it is interpreted as an etiologic conclusion rather than a descriptive label. Evaluation should distinguish PAP adherence from treatment efficacy and symptomatic recovery, determine whether the complaint reflects sleepiness rather than fatigue or apathy, and consider alternative contributors including insufficient sleep, circadian misalignment, medication or substance effects, medical and psychiatric conditions, coexisting sleep disorders, and central disorders of hypersomnolence. Additional testing should be guided by a defined clinical question, and wake-promoting therapy should follow rather than replace diagnostic reassessment. Persistent sleepiness after effective PAP describes an observation, not an etiology, and deserves independent clinical evaluation.

## Editorial

Positive airway pressure (PAP) therapy often improves excessive daytime sleepiness in patients with obstructive sleep apnea (OSA), but effective control of obstructive respiratory events does not invariably restore normal daytime alertness. Even when PAP adequately controls sleep-disordered breathing, clinically important sleepiness may persist. When this occurs, the symptom deserves its own clinical explanation rather than automatic attribution to OSA. The commonly used term “residual excessive daytime sleepiness” is clinically convenient, but it can become misleading when a descriptive label is interpreted as an etiologic conclusion. Persistent sleepiness after PAP therapy describes what the patient is experiencing; it does not establish why the patient remains sleepy. The symptom may still be related to OSA, but it may also reflect another contributor or a coexisting disorder. The term “residual” should therefore not prematurely close the differential diagnosis.

A useful starting point is to distinguish PAP adherence, PAP efficacy, and clinical recovery. These concepts overlap, but they are not synonymous. Weaver et al. demonstrated a dose-response relationship between nightly continuous positive airway pressure (CPAP) use and improvement in subjective sleepiness, objective sleep propensity, and daily functioning, with different amounts of CPAP use associated with normalization across these outcomes [1]. A patient may therefore use PAP consistently without achieving equivalent recovery across every clinical domain. Adherence alone does not establish efficacy, and efficacy alone does not establish symptomatic recovery.

Persistent sleepiness after PAP should therefore prompt a renewed diagnostic question rather than simply be regarded as an extension of the original OSA diagnosis. Pépin et al. reported excessive daytime sleepiness in 60 of 502 CPAP-treated patients (12.0%) at one-year follow-up. After excluding several important competing conditions, specifically restless legs syndrome, major depressive disorder, and narcolepsy, the estimated prevalence decreased to 30 of 502 patients (6.0%) [2]. This reduction illustrates why diagnostic reassessment matters: some apparently residual sleepiness may reflect identifiable conditions requiring management distinct from OSA itself.

Timing further complicates interpretation. In the European Sleep Apnea Database, Bonsignore et al. found that excessive daytime sleepiness was most prevalent among patients assessed during the first three months of CPAP treatment and less prevalent among those assessed later [3]. Residual OSA and insufficient CPAP adherence occurred at similar frequencies among patients with and without excessive daytime sleepiness at follow-up [3]. These findings support caution against interpreting sleepiness identified early in treatment as a stable long-term phenotype. Clinically significant sleepiness nevertheless warrants continued assessment rather than passive observation.

Before sleepiness is attributed to treated OSA, the symptom itself should be characterized. Fatigue and apathy may be mistaken for excessive daytime sleepiness, although these experiences are not equivalent [4,5]. Contemporary consensus and practice guidance therefore emphasize careful assessment of sleepiness as a distinct clinical symptom [4,5]. Subjective scales can help quantify symptom burden, but a questionnaire score should not become the diagnosis.

Once the complaint has been characterized as sleepiness, the next step is to confirm that OSA treatment is genuinely effective rather than merely documented as adherent. PAP use should be interpreted in relation to the patient’s habitual sleep period, and available treatment data should be reviewed to determine whether sleep-disordered breathing is adequately controlled. If OSA is effectively treated, the differential diagnosis should broaden to consider insufficient sleep, circadian misalignment, medication or substance effects, medical and psychiatric conditions, and coexisting sleep disorders [4,5]. Treated OSA remains part of the clinical context, but its presence should not prematurely narrow the differential diagnosis.

When sleepiness remains unexplained after adequate sleep opportunity and effective OSA treatment have been established, additional testing should be selected to answer a defined clinical question rather than ordered reflexively. Central disorders of hypersomnolence should be considered when the clinical history is suggestive, with objective testing used selectively when it is likely to clarify diagnosis or management [4,5]. Wake-promoting therapy may be appropriate for selected patients, but pharmacologic treatment should follow rather than replace this diagnostic process. Improving alertness can be clinically valuable; it does not establish why the patient remained sleepy.

Effective PAP therapy should therefore be regarded as completion of one therapeutic task rather than completion of the entire clinical evaluation. PAP addresses sleep-disordered breathing; persistent sleepiness raises a separate question about daytime alertness and function. These problems are frequently related, but control of one should not be assumed to establish resolution of the other. When clinically important sleepiness persists despite effective PAP therapy, the question should shift from whether the airway disorder has been treated to why the patient remains sleepy. Persistent sleepiness after PAP describes an observation, not an etiology. Adherence alone does not establish efficacy. Efficacy alone does not establish symptomatic recovery. Persistent sleepiness therefore deserves independent clinical evaluation.
